# A Study on Immersion and Presence of a Portable Hand Haptic System for Immersive Virtual Reality

**DOI:** 10.3390/s17051141

**Published:** 2017-05-17

**Authors:** Mingyu Kim, Changyu Jeon, Jinmo Kim

**Affiliations:** Department of Software, Catholic University of Pusan, Busan 46252, Korea; kmg2917@naver.com (M.K.); jcg1993@naver.com (C.J.)

**Keywords:** immersive virtual reality, hand haptic system, Arduino-based sensor, interaction, immersion, presence

## Abstract

This paper proposes a portable hand haptic system using Leap Motion as a haptic interface that can be used in various virtual reality (VR) applications. The proposed hand haptic system was designed as an Arduino-based sensor architecture to enable a variety of tactile senses at low cost, and is also equipped with a portable wristband. As a haptic system designed for tactile feedback, the proposed system first identifies the left and right hands and then sends tactile senses (vibration and heat) to each fingertip (thumb and index finger). It is incorporated into a wearable band-type system, making its use easy and convenient. Next, hand motion is accurately captured using the sensor of the hand tracking system and is used for virtual object control, thus achieving interaction that enhances immersion. A VR application was designed with the purpose of testing the immersion and presence aspects of the proposed system. Lastly, technical and statistical tests were carried out to assess whether the proposed haptic system can provide a new immersive presence to users. According to the results of the presence questionnaire and the simulator sickness questionnaire, we confirmed that the proposed hand haptic system, in comparison to the existing interaction that uses only the hand tracking system, provided greater presence and a more immersive environment in the virtual reality.

## 1. Introduction

Various virtual reality (VR) applications within different areas aim to provide users with an immersive experience. Although virtual experience has been a subject of in-depth and intense research, the demand remains for an interactive system with an even greater immersion and sense of reality through feedback that can satisfy the five senses, including visual, auditory, and tactile, to enhance the user’s sense of being present in a VR environment. Immersion and presence are described as follows. Through user’s five senses, immersion allows users to experience where they are, whom they are with, and what they are doing as if it is a real experience [1]. In addition, immersion is defined as the extent of engagement a user experiences; the user expresses various emotions in a virtual space, and delivers them to the virtual environment [2]. Here, the concept of presence refers to a phenomenon where users act and feel as if they are “being there” in the virtual world created by computer displays [3,4,5]. Consequentially, immersion is a term used for describing the technology that can give rise to presence [6]. If the VR environment is not used properly, it may cause symptoms such as headaches, dizziness, and nausea. This is called VR sickness [7]. This has led to the development and distribution of VR technology and devices that support visual, auditory, and tactile senses within the VR environment. Recently, there have also been studies on multimodality, which enhances the sense of immersion through interaction via more than two senses (e.g., vision and sound, or touch and sound) [8,9,10]. Similarly, there has been research on enhancing the sense of presence through sensory and sound feedback from the user wearing haptic shoes as their foot touches the ground [11]. In addition, studies were conducted to design and experiment on display parameters, visual realism, haptics, etc. as factors influencing presence in virtual environments [4] and to investigate the process of changing behaviors and perceptions depending on the environment and conditions of users in VR [12]. Because of the lack of technology or devices that can detect other senses such as taste, smell, and touch and the lack of a system that can be easily used, this area still requires more research.

Without a haptic system, interactions with objects in the VR environment can lead to a gap between the actual and virtual realities; therefore, feedback from such interactions with objects through a haptic system is crucial in order to accurately express the interaction between the virtual object and reality [13]. This is an essential step in enhancing the sense of presence and immersion in VR applications. Initial studies focused on VR gloves or tables that offer haptic feedback; the scope of research has now expanded to include the accurate detection of face and hand motions and the offering of force feedback. However, the scope of expression for these devices is limited, costly, and complex; therefore, they cannot be commercialized for the public [14]. In particular, one of the problems is that the feedback of this type of system lacks diversity. To overcome this problem, researchers are investigating a redirection method of providing visual information using real and virtual objects at the same time so that users will feel as if they are touching the virtual object, as a way to enhance the presence [15]. Such research on passive VR has evolved to include studies using real props [16]. However, this also has limitations of conditions, environment, and expense. Recently, with regards to the wearable tactile interface, a study has been conducted to optimize device configuration and implement tactile feedback on fingertips with a simple structure and high performance [17]. In other words, the study is intended at considering aspects ranging from accuracy to applicability of the haptic system. Further, this study analyzes the satisfaction of the feedback when using a wearable haptic device by presenting various situations to participants in the virtual environment [18].

Therefore, this study proposes a portable hand haptic system that can be used for various VR applications and that can provide diverse feedback at low cost by focusing on tactile senses. The hand tracking system detects hand motions and expresses them in the VR environment for accurate interaction with virtual objects. Then, we conduct experiments through a survey for the participants and determine the satisfaction and applicability of the proposed portable hand haptic system.

The proposed portable hand haptic system makes the following contributions:

- A portable system that effectively provides a diverse haptic reaction at low cost was implemented to ensure that the hand haptic system can easily and conveniently used by anyone.
- Technical and statistical experiments were conducted to determine whether the proposed system provides a higher presence and greater immersion for users in immersive virtual reality.

**Figure 1:**
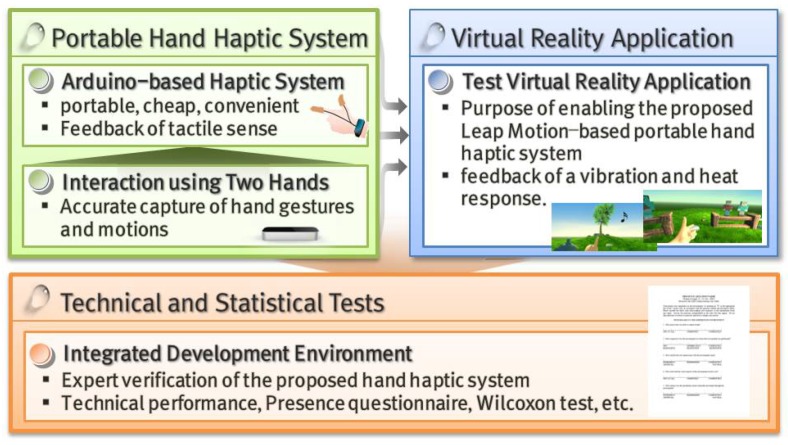
Overall structure and flow of this study.

Figure 1 shows the overall structure of this study.

Section 2 discusses the research on conventional haptic systems, Section 3 explains the proposed portable hand haptic system in detail, and Section 4 describes the test elements necessary for the verification of the proposed system. In Section 5, the technical and psychological effects of interaction with the haptic system on the user are explained, including the test results. The limitations of this study are mentioned in Section 6, and lastly, Section 7 presents final conclusions and directions for future research.

## 2. Related Works

Research on the interaction between human and computer is still being carried out in various ways. With the development and distribution of various applications that utilize many VR devices (e.g., head mounted displays, HMDs), research on interaction is receiving more attention. Although a majority of 3D applications control methods, 2D input devices such as mouses and keyboards are also widely used. However, when the concept of 3D is expanded into immersive VR, such input devices face limitations. Therefore, there have been studies on input devices and technologies that can provide interaction with greater immersion, which has led to research on haptic interaction and feedback [19]. Haptic systems in particular provide an experience similar to actual reality through tactile and force feedback. More specifically, haptic interfaces provide a sense of touch by using electrical actuators, and force feedback systems have been designed to resist or constrain users’ behavior [20,21].

Among the diverse studies on haptic interfaces, Yano et al. [22] proposed a hand-held haptic device by which users can touch a virtual object directly with their fingers. Dorjgotov et al. [23] studied an immersive granular material system that includes haptic feedback. Meanwhile, Danieau et al. [24] proposed HapSeat, a system that enables simulation and actuation of head and hand movements at low cost. There have also been studies on the expression of feedback-enhanced user interaction for immersion in virtual environments that included a visual/haptic interface [25]. Although most of the initial haptic interface research has focused on hands, it has now expanded to include interactions with other body parts. With the increased interest in multimodality in particular, Stefania et al. [11] used VR haptic shoes and sound synthesis to study the identification of the virtual ground. As such, researchers have been actively studying the effects of audio-tactile interaction related to multimodality [26,27]. Research on wearable haptic devices has been advanced based on these studies. Leonardis et al. [28] proposed a 3-RSR Haptic Wearable Device, composed of asymmetrical three revolute-spherical-revolute, that can control the modulating contact forces at the tip of the finger. Prattichizzo et al. [29] proposed a new three degree of freedom (3-DOF) wearable haptic interface that can directly apply a force vector to the finger. Schorr et al. [30] proposed a wearable fingertip haptic device that is capable of making and breaking contact with the finger pad. Bianchi et al. [31] examined the Wearable Fabric Yielding Display (W-FYD), which is a fabric-based haptic display for multiple queue transfer, that can be worn on the finger. In addition, Benali-Khoudja et al. [32] conducted thermal modeling of the thermal transfer that occurs between the finger skin and the explored surface. However, such haptic interfaces have limitations in terms of cost and conditions, and thus utilizing them in other VR applications is difficult. A study by Scheggi et al. [33] recently analyzed immersive virtual reality with a similar goal to that of our study, using a Leap Motion controller and wearable tactile devices. However, their study only presented the direction for future research and did not show specific outcomes.

Prior to the provision of feedback to the user from physical responses that occur in the virtual environment, accurate detection of motions is necessary. Recently, there have been a variety of investigations into motion platforms that are appropriate for VR—ones that capture users’ expressions, gestures, and movements to enhance interaction presence. Li et al. [34] conducted research on the detection of facial displays by attaching a depth sensor to an HMD, which is a VR display device. The hand is the part of the body that is used the most to interact with other objects—not only in reality, but also in the virtual world. For this reason, there have been studies on the capture of hand and finger movements by joints to reflect them in the virtual space. Zhao et al. [35] accurately measured joint movement by combining a hand motion capture system based on optical markers and a Kinect camera. Meanwhile, Metcalf et al. [36] proposed a surface marker-based optical motion capture system and conducted research on capturing movement accurately. Likewise, there have been various studies on attaching markers directly on hands and accurately capturing hand motions via camera [37,38]. In order to apply such research to VR applications, however, one must consider the convertibility to other devices (e.g., HMDs). Therefore, applying hand motion capture research to the actual design of VR applications is very rare.

Leap Motion expresses free hand motions within 3D space, and has recently been developed. This technology also expresses hand and finger motions virtually by accurately capturing them, and thus researchers are actively studying the possibility of its application in content development by adapting it for other VR technologies at low cost. Among the areas of research related to this is a study on producing and verifying signatures using the tip of the index finger when the user imitates a writing motion using his or her finger [39], and a study on applying the realistic manipulation of surgical instruments using the hands [40]. However, motion-sensing device can only detect hand motions and is not equipped with the ability to provide tactile feedback during interaction with a virtual object. Therefore, combining these hand motion-sensing device—which enables the elaborate detection of hand and finger motions in VR—with a haptic system which can deliver various tactile responses in simple format at low cost is expected to be more useful for VR application.

To this end, this research aims to propose a portable hand haptic system using Leap Motion that includes various tactile responses and that can provide users with an immersive VR environment and experience at a low cost.

## 3. Portable Hand Haptic System

### 3.1. System Overview

**Figure 2:**
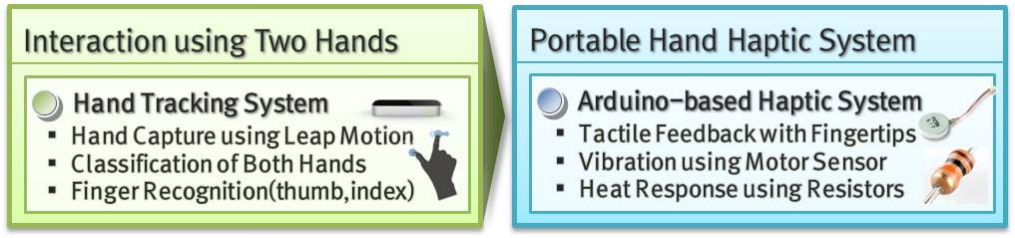
Overall outline of the portable hand haptic system using Leap Motion.

This research proposes a portable haptic system that can provide various tactile responses in order to enhance interactive immersion within VR applications using hands. This system can be divided into two main components. First, there is a haptic system, which can deliver tactile senses to the hands according to the situations users are in. The crucial aspect of this system is that it must be a simple and portable system that can express a variety of tactile senses at a low cost. Therefore, in this study, the sensors are controlled using Arduino [41], and a band-type structure is proposed so that tactile modules can be easily attached, enabling the system to be easily used for VR applications. Secondly, prior to providing tactile haptic feedback, it is essential to accurately detect hand gestures and motions, and to this end, Leap Motion device [42] is utilized in this research. With hand motion-sensing device, the user’s hand gestures and motions are accurately reproduced in the virtual environment, and the haptic system provides feedback that represents an interactive physical response. Figure 2 shows the overall flow.

### 3.2. Arduino-Based Haptic System

In order to design a portable hand haptic system that can express a variety of tactile senses at a low cost, using an Arduino is most beneficial, as it is small in size, cheap, and easily convertible. Therefore, in this study, an Arduino nano board was used to design all of the systems. The reason for using a nano board is that the proposed system must be made into a type of wristband in order for it to be portable, and the system was designed as band-type tactile sensors to be worn on the fingertips. In short, the main board that handles all tactile senses delivered to the hand was made into a wristband, and each tactile sensor into a wearable band for the fingertip.

First, this study proposes two sensors with vibration using vibrating motors and heat response with a resistor to deliver tactile senses to the fingertips. Each sensor is made into one band; it is portable, and users can wear the bands corresponding to the applications. It is important that the system is not limited to only two sensors but is Arduino-based, which means that more sensors can be added. The aim of the research is to design a portable system architecture that can react to tactile senses using various sensors that can be connected to the Arduino. As a system, the prototype is designed to be applied only to the thumb and index finger.

**Figure 3:**
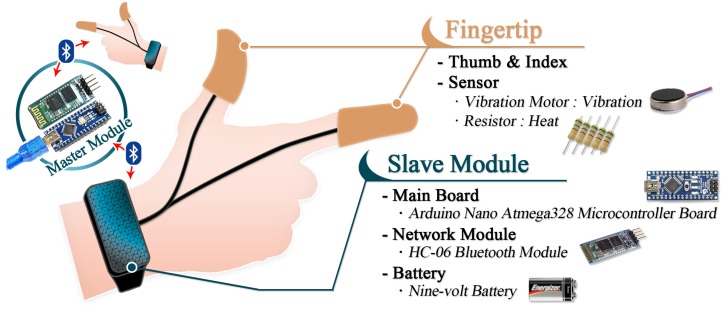
Hardware configuration of Arduino-based portable hand haptic system.

Next, as the wristband is a control board for the modules that are connected to the fingertips of the thumb and index finger, it manages all tactile senses delivered to one hand. Since the proposed hand haptic system processes the tactile reactions for each hand separately, the user must wear two wristbands. This calls for a master board in the center that can control all signals delivered to each hand. To this end, this study designates each wristband as a slave and the main board that controls the two hands as the master when arranging the process for data transmission and reception. Here, the data exchange is processed via Bluetooth. Therefore, the wristband board consists of a Bluetooth module on the Arduino nano board and a battery for the power supply. Figure 3 shows the hardware configuration of the Arduino-based portable hand haptic system.

**Figure 4:**
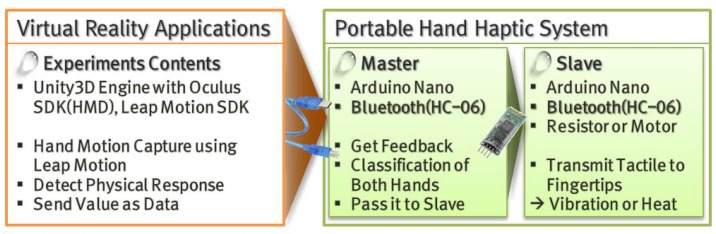
Process of data transmission between virtual reality (VR) application and proposed hand haptic system. HMD: head mounted display.

When a physical response occurs within an interaction in a VR application, the process of delivering an appropriate tactile sense to the fingertips must be defined in detail. To this end, the study used a suitable environment created through a combination of serial and Bluetooth communication methods. As mentioned previously, the Arduino-based hand haptic system designates the two hands as slaves and the central controller as master and uses Bluetooth to transmit data between the two points. Here, the slave handles the sending of signals to the fingertips in accordance with the currently connected tactile sensor. Therefore, the master has a more important role. The central role of the master is to receive the physical response that occurs in the VR application and to send the signal to the correct slave by distinguishing the left and right hands. Next comes the data transmission between the VR environment and the master board, which is processed quickly by using the serial port directly. Here, the VR application is also granted direct access to each hand’s wristband slave board. In this case, however, the VR application must go to the trouble of designing a separate communication system for each hand. Therefore, in this study, a communication flow was designed as shown in Figure 4 to enable the VR application developer to make effective use of the proposed haptic system.

### 3.3. Interaction

For the user to feedback a physical reaction in an interaction using the hands in VR space, the accurate capture of hand gestures and motions is essential. However, if the hand haptic system (e.g., VR glove) detects hand joint motions and includes tactile responses, its price will increase and its processing will become too complex [43]. Therefore, this paper proposes a portable haptic system that can deal with tactile hand senses, in which hand motions are accurately detected using the hand tracking system that is mounted on HMD. Consisting of two infrared cameras and an infrared light source, hand motion-sensing device detects motion and simulates it in 3D virtual space when there are hand motions in front of the infrared sensors. As this study investigates the response on the thumb and index finger, finger movement detection and gesture recognition are also carried out, focusing on these two fingers. Algorithm 1 summarizes the finger recognition process that is appropriate for the haptic system proposed in this study based on the hand capture, recognition, and finger classification modules provided in Leap Motion software development kits (SDK). it represents the process of distinguishing the left or right hand as the hand for which movement has been detected and also the process of distinguishing each digit and recognizing the thumb and index finger either separately or together.

**Algorithm 1** Finger recognition based on Leap Motion.
1:frame ← captured scene information.2:**procedure** hand recognition(frame)3:    Finger OneHand = frame.Hands[0].Fingers4:    save recognized finger information of 0*^th^* hand.5:    **if** frame.Hands[0].IsRight ⩵ true **then** ← right hand recognition.6:        **if** OneHand[0].IsExtended ⩵ true **then**7:           right hand thumb recognition.8:        **end if**9:        **if** OneHand[1].IsExtended ⩵ true **then**10:           right hand index finger recognition.11:        **end if**12:    **else if** frame.Hands[0].IsLeft ⩵ true **then** ← left hand recognition.13:        **if** OneHand[0].IsExtended ⩵ true **then**14:           left hand thumb recognition.15:        **end if**16:        **if** OneHand[1].IsExtended ⩵ true **then**17:           left hand index finger recognition.18:        **end if**19:    **end if**20:
**end procedure**



**Figure 5:**
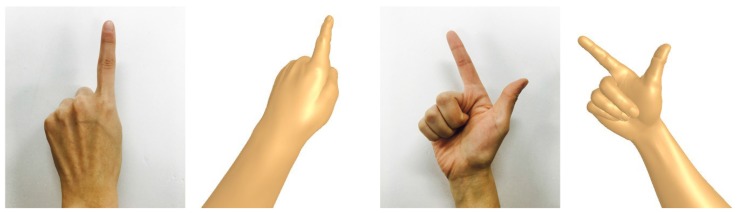
Hand gesture recognition process of proposed hand haptic system.

Figure 5 shows the results of expressing gestures in 3D virtual space in accordance with the tactile senses that are handled in the proposed haptic system focusing on the index finger and thumb.

## 4. Virtual Reality Applications

The test applications were designed for allowing the proposed portable hand haptic system to provide users with interaction that enhances immersion in a VR application. Applying the proposed haptic system to already commercialized VR applications is the objective. Because of licensing issues and limitations in finding the most ideal application, an optimized application was designed for this study. The VR application must have a scene that corresponds to the two types of tactile sensors of the haptic system.

**Figure 6:**
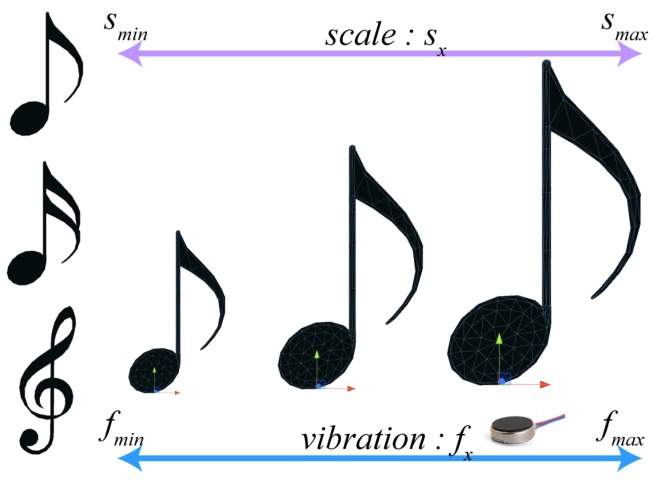
Set of virtual reality (VR) application objects for vibrational tactile feedback response.

The first scene was designed using the playing of music as the theme in order to test the interaction of sending the feedback of a vibrating tactile response. In the application, objects of a certain size are randomly created and moved toward the user. When the user touches the object using the thumbs and index fingers of both hands, the object is bounced off and sends tactile senses. The intensity of the tactile sense is set to be in proportion with the size of the virtual object, and the user is asked to send a feedback response that is equivalent to the tactile sense (Equation (1)). Figure 6 shows the virtual objects and vibration sensor range for the first scene.

(1) $$f_{x}=f_{min}+\frac{\left(f_{max}-f_{min}\right)}{\left(s_{max}-s_{min}\right)}*\left(s_{x}-s_{min}\right)$$

Here, $s_{min}$ and $s_{max}$ are the minimum and maximum size of the VR objects, and $f_{min}$ and $f_{max}$ are the minimum and maximum intensity of vibration delivered to the fingertips. $f_{min}$ and $f_{max}$ are set as 128 and 255, respectively, as only an intensity of over 128 can be felt by the hand and the maximum range of the vibration sensor is 255. When object size $s_{x}$ is confirmed, the vibration intensity $f_{x}$ that is delivered to the fingertips can be calculated using Equation (1).

**Figure 7:**
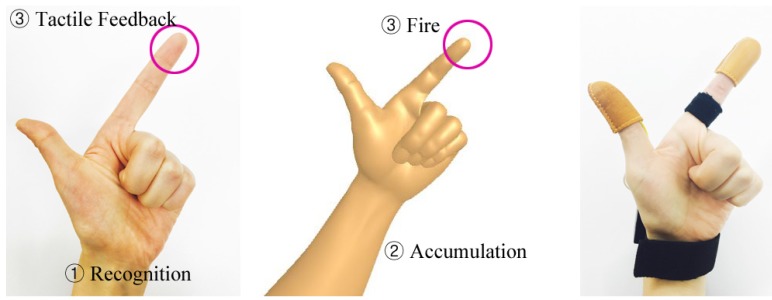
VR application hand gesture processing for heat tactile reaction .

The second scene is one that is appropriate for a feeling using the heat sensor. For a heat sense, a resistor is used in this study, which is the accumulated heat that is delivered as opposed to the heat that occurs with an instantaneous reaction. Therefore, an application that can effectively reflect such a characteristic must be designed. The second scene features a hand gesture that requires the extension of only the thumb and index finger, and the gesture is recognized as it matches that of a hand holding a pistol. After a certain time, the bullet is fired from the end of the index finger. At the moment of firing the bullet, heat is accumulated in the fingertip in order to deliver a feedback response that makes it seem as if a bullet has actually been fired from the fingertip. What is crucial here is that the time the heat is delivered to the fingertip through the heat sensor must match the time the pistol is fired in virtual space. Therefore, the accumulation time of the haptic system’s heat sensor must be calculated and applied to the VR application. Figure 7 shows the process of recognizing and delivering the heat reaction in the second scene, and Algorithm 2 is a more detailed explanation of this process.

**Algorithm 2** Hand recognition and heat response method.
1:$H_{g}\leftarrow$ finger gesture.2:state = 0 ← current state (0: initial, 1: pistol gesture, 2: heat accumulation completion).3:$t_{heat}\leftarrow$ heat reaction accumulation threshold time.4:**procedure** heat response feedback($H_{g}$, state, $t_{heat}$)5:    **if** $H_{g}$ is pistol gesture **And** state ⩵ 0 **then**6:        state = 17:        signal delivery to heat sensor.8:    **end if**9:    **while** $H_{g}$ is pistol gesture **And** state ⩵ 1 **do**10:        t← heat accumulating time calculation.11:        **if** $t\geq t_{heat}$ **then**12:           state = 213:           t=0← time initialization.14:        **end if**15:    **end while**16:    **if** state ⩵ 1 **And** $H_{g}$ is not pistol gesture **then**17:        state = 018:        t=019:    **else if** state ⩵ 2 **then**20:        firing bullet from fingertip.21:        heat sensor block.22:        state = 023:    **end if**24:
**end procedure**



**Figure 8:**
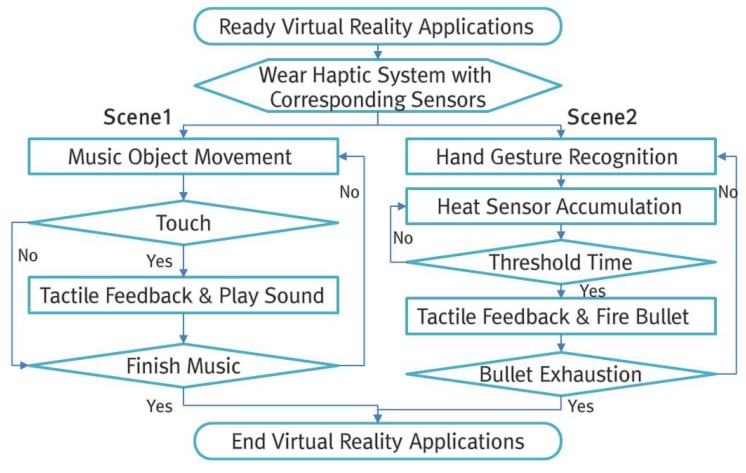
Process flow of VR application for proposed hand haptic system.

Figure 8 shows the overall flow of the VR application that consists of these two scenes.

## 5. Experimental Results and Analysis

The proposed portable hand haptic system function was implemented using Arduino Sketch v.1.6.12 (Interaction Design Institute Ivrea (IDII), Ivrea, Italy), and the VR application for testing was designed using Unity 3D 5.3.4f1 (Unity Technologies, San Francisco, CA, USA), Oculus SDK (ovr_unity_utilities 1.3.2, Oculus, City of Irvine, CA, USA), and Leap Motion SDK v4.1.4 (Leap Motion, Inc., San Francisco, CA, USA). The specifications of the PC used for system design and experiments were an Intel Core i7-6700 (Intel Corporation, Santa Clara, CA, USA), 16 GB random access memory (RAM), GeForce GTX 1080 GPU (NVIDIA, Santa Clara, CA, USA). One of the objectives of the proposed portable hand haptic system was low cost. In this study, the system was designed using Arduino, and the total cost of making a band-type system for two hands was *$*32.79 (nano board: *$*3.93 × 3, vibration motor and resistor: *$*0.84 × 4, Bluetooth: *$*4.45 × 3, cable: *$*0.61, case and band: *$*1.84 × 2), and thus it can be confirmed that the system was made for a low cost.

**Figure 9:**
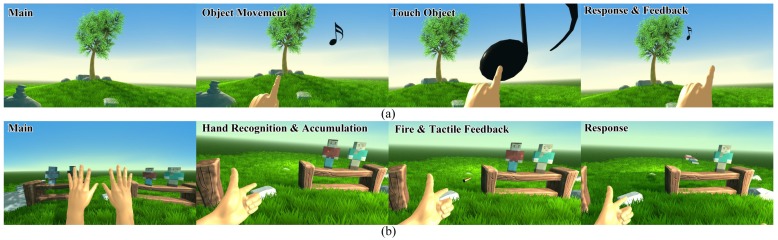
Proposed VR application design results: (**a**) virtual scene for vibration sensor; (**b**) virtual scene for heat sensor.

First, the following are the results of designing a VR application with the aim of testing and confirming that the proposed portable hand haptic system provides users with a realistic sense of immersion and presence. As shown in Figure 9, an application with two scenes was developed, and it was executed according to the flow in Figure 8. A VR application must consider crucial technical aspects, including the frames per second (fps) and the total number of polygons (k, kilobytes) in a rendering scene. As for the PC-based VR development environment, the frames per second must not exceed 75, and the number of polygons must not exceed 1024–2048k in order to prevent technical issues such as display delay phenomena or a misrecognition that can lead to VR sickness. In the proposed VR application, it was confirmed that the number of polygons from the camera perspective for Scenes 1 and 2, respectively, was minimum 188.5k and 269.5k and maximum 350.2k and 379.2k, and thus an average of 249.4k and 298.3k was maintained. Moreover, the number of frames per second (fps) stood at minimum 85.2 and 87.5 and maximum 98.7 and 127.1, with an average of 90.1 and 105.4; therefore, it was confirmed that the application as designed did not have any hardware-related issues.

**Figure 10:**
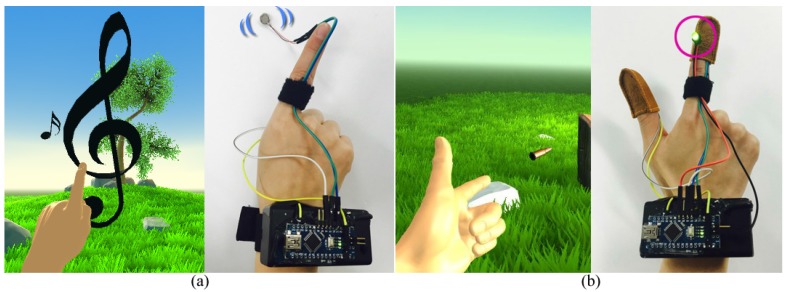
Physical feedback response results through interaction between proposed portable hand haptic system and VR application: (**a**) vibration after touch; (**b**) expression of heat through light emitting diode (LED).

Next, the experiment confirmed normal operation with a correspondence of interaction between the proposed hand haptic system and the designed VR application. The application is composed of two scenes that react to vibration and heat, respectively. In the first scene, when the moving virtual object is touched with a fingertip, vibration is delivered along with the defined scale sound (Figure 10a). When a user touches all objects without missing any, the application was designed to play a children’s song that was defined in advance. In the second scene, when the user makes a defined gesture using his or her thumb and index finger, the heat sensor accumulates the heat, and when heat is felt on the user’s fingertip, the bullet is fired. Because of difficulties in expressing heat in video, the results were confirmed by attaching an LED to the heat sensors (Figure 10b). For both of the scenes, it was confirmed that the application was interacting with the user in correspondence with the proposed haptic system.

**Figure 11:**
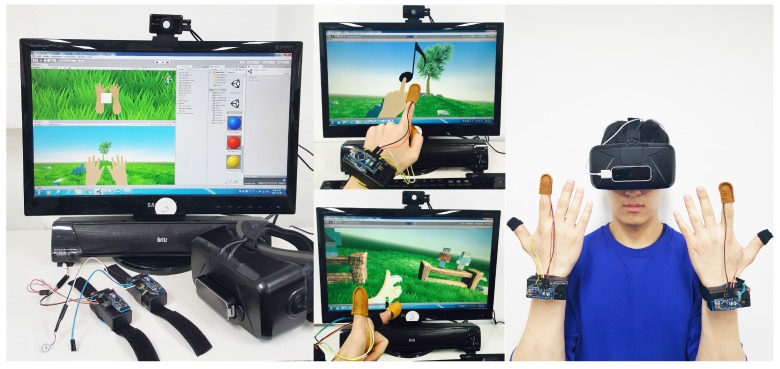
Experiment environment with general users as targets.

Lastly, a survey was conducted to assess user immersion and presence for the portable hand haptic system. We investigated whether the hand haptic system can provide more immersive environments than the existing interaction system, which uses only the hand tracking system. And we had to check whether the proposed haptic system and virtual reality application enhances user satisfaction of presence by providing users with an experience that is similar to reality. A total of 21 people were randomly selected between the ages of 21 and 35, among whom nine were women and the remaining twelve were men. As the VR application designed for the experiment only involved the hands, an environment was created so that users could sit to experience the application. Figure 11 shows the experiment environment for the VR application using the proposed haptic system. This study used the Oculus Rift development kit (DK) 2 (Oculus, City of Irvine, CA, USA) as an HMD to send the VR scenes to participants, and the Leap Motion (Leap Motion, Inc., San Francisco, CA, USA) was attached to the front of the HMD. Participants wore the HMD on their head and the proposed hand haptic system on their hands, as shown in Figure 11. Then, the three-dimensional visual information was transmitted to users through the two lenses of the HMD, and the defined tactical response was transmitted through the haptic system. In the test, interaction using the hand tracking system (H) only was compared with interaction using the proposed portable hand haptic system (S) to analyze immersion and presence for each of them. At this point, half of the participants (4 females, 6 males) experience the first VR application, that consists of two scenes, through interaction using only the hand tracking system. Then, they experience the first scene while wearing vibration sensors and the second scene while wearing heat sensors. The remaining half of the participants (5 females, 6 males) proceeds in reverse order.

The first test consisted of elements that aimed to analyze sense of immersion provided to users by the proposed portable hand haptic system within the VR application. Namely, it is confirmed whether the proposed portable hand haptic system is suitable as a technology to provide immersion in a virtual environment. Table 1 shows the answers to five questions as the result of the comparison experiment. First, it was asked whether the VR application proposed in this research was appropriate for testing the proposed haptic system, and the answers were recorded. Participants recorded a score between 1 and 5 based on 3 (Appropriate), and were allowed to enter a rating of up to 5 if they felt the highest amount of satisfaction. Both scenes received high average scores (mean 4.1, 3.8) close to 4, which means that the application was appropriate. Here, the standard deviation was calculated as 0.61 and 0.59, respectively. Next, the tests for vibration and heat sensors were conducted separately. (b)–(c) give the analysis results for sense of immersion that the vibration sensor provides users, and it was first assessed whether the time the virtual object was touched and the time the vibration was delivered to the fingertip coincided. With an average of 2.94 and standard deviation (SD) of 0.22, which is a high score, it was confirmed that the majority of users recognized the vibration at the time of touch without any significant time error margins from the reference value. The immersion that the vibration sensor provides users was also analyzed, and the experiment was carried out so that the result could be compared with the interaction of general applications that use hand motion-sensing device only. Assuming the sense of touching an actual object, the proposed haptic system was confirmed to deliver a relatively high level of satisfaction (mean 2.73, 4.43). Then, the standard deviation was calculated as 0.94 and 0.52, respectively. The same test method was applied to the heat sensor; it was asked whether the point of feeling peak heat coincided with the time the bullet fired. Although the reaction was slower than for vibration, the results (mean 2.23 (SD 0.44)) were not unnatural for the users. It was also confirmed that there were differences in the values due to the difference in the intensity of heat felt by each participant. Because of this, immersion was found to have a high level of satisfaction among participants who accurately recognized heat, whereas those participants who did not accurately recognize heat answered that their immersion was interrupted. Therefore, the scores were relatively higher with a small difference in value (mean 3.09, 3.32). At this point, the standard deviation was calculated as 0.69 and 0.76, respectively.

For statistical analysis, we conducted the Wilcoxon test on the hand tracking system and hand haptic systems based on the survey data. The Wilcoxon test results were calculated using the scores recorded by the participants in questions (c) and (e). The experiment was conducted by testing immersion in the case of the vibration haptic sense and in the case of the heat reaction, as well as by providing a comprehensive analysis that combined the two cases. In the test results, the *p*-value of immersion in the case of the vibration haptic sense was $5.7605\times 10^{-5}$, which is smaller than the significance level of 0.05. Thus, we can reject the null hypothesis. However, the *p*-value was $9.2421\times 10^{-1}$ in the case of the heat reaction, so we could not reject the null hypothesis. Although the proposed haptic system provides greater immersion in terms of the vibration haptic sense, it was shown that a difference of perception was important in the case of the heat reaction. With regard to the comprehensive analysis, we can support the hypothesis that the portable haptic system with a simple structure can provide a better immersion with a *p*-value of $3.5608\times 10^{-4}$.

For the second test, we wanted a survey that could more objectively investigate whether the virtual reality environment of the proposed hand haptic system can provide higher presence to users. In other words, it is confirmed whether the user actually felt presence through the proposed portable hand haptic system. Thus, this study adopted the presence questionnaire suggested by Witmer et al. [44] to implement in an identical environment as the previous experiment group. This questionnaire consists of a total of 24 items. It can comprehensively analyze the presence experienced by the users can in the virtual space. Moreover, it is capable of specific analysis by dividing the items into realism (items 3, 4, 5, 6, 7, 10, and 13), the possibility of act (items 1, 2, 8, and 9), the quality of the interface (items 14, 17, and 18), the possibility of examine (items 11, 12, and 19), the self-evaluation of performance (items 15 and 16), the sounds (items 20, 21, and 22), and the haptic (items 23 and 24). The participants input scores between one and seven for each item. Here, higher value means higher satisfaction in terms of presence in most cases. Table 2 shows that the virtual reality environment in which the proposed hand haptic system is implemented produces greater overall satisfaction (141.0 < 148.2, 150.9) than the method that uses only the hand tracking system.

With regard to the heat reaction, the difference in value was relatively small compared to the haptic reaction. Nevertheless, the proposed system still provided greater overall presence than the method that used only the hand tracking system. In particular, based on the results for the possibility of act, the quality of the interface, and the haptic items, it was confirmed that the proposed haptic system, in comparison to the existing method that simply perceives hand movement, was better at enhancing presence when interacting with virtual objects in virtual reality. Thus, we confirmed that the proposed haptic system provides higher presence and immersion to induce concentration during the interaction.

Presence also performed the Wilcoxon test based on all scores the 24 items entered by participants, in order to obtain meaningful statistical results, such as immersion. The *p*-value was $4.9203\times 10^{-3}$ and $5.0050\times 10^{-3}$, respectively, when the results of interaction using only the hand tracking system and each of haptic systems consisting of heat sensors and vibration sensors were compared. Consequentially, it can be statistically confirmed that the proposed portable hand haptic systems provides higher presence to users in VR.

## 6. Limitations

The purpose of the proposed portable hand haptic system is to present a new tactile system that can be carried around easily for use as a VR device like Google Cardboard. Because of the difference in objectives, however, the drawback of this system is that it looks much simpler than a conventional haptic system, which is very complex in structure [45,46]. In addition, commercial hand motion-sensing device was used in this study for capturing hand gestures and motion. This can conversely be interpreted as meaning that the proposed haptic system has limitations, in that it needs the hand tracking system such as Leap Motion. If the tactile sensors to be added cannot be provided in band form like the two proposed sensors, the structure of the entire system could likely be transformed. It also has limitations in generalizing the reaction, since users using the heat sensor through the resistor feel the heat at different times. Lastly, the objective conclusions that can be drawn using the proposed VR application are limited; the proposed haptic system must be coupled with commercial applications in order to obtain more objective verification.

## 7. Conclusions and Future Work

This study proposed a portable haptic system that anyone can use to easily deliver a variety of tactile feedback at low cost. By focusing on hand tactility, the proposed haptic system is designed to provide a haptic interface that can be applied to various VR applications. To this end, an Arduino-based hand haptic system that provides feedback through fingertips was designed. In order to measure tactile reactions separately for each hand, the proposed haptic system was designed in a master–slave configuration. Here, the master receives the physical reaction that occurs in the VR application and delivers the corresponding tactile sense to the appropriate hand and finger by distinguishing left and right hands. Using the information delivered from the master, the slave transmits the corresponding reaction to the connected tactile sensor. The prototype of the proposed hand haptic system considers two types of tactile senses: vibration and heat reaction. In order to capture hand gestures and motion necessary for tactile feedback using the hands, this study proposed the use of hand tracking system for the interaction. This is because capturing tactile senses and hand motions and processing them within the haptic system can become complicated and expensive. The hand detected through motion-sensing device can be identified as left or right, and the movement of finger joints can also be distinguished. Since thumbs and index fingers were used in this research, gestures and motion were accurately detected by focusing on thumbs and index fingers. The hand tracking system accurately detects the user’s hand motion and processes interactions in virtual space. When a physical reaction occurs during this process, the corresponding tactile sense is transmitted via the proposed haptic system. Lastly, an application was designed in order to accurately test the portable hand haptic system for immersion and presence. Since the proposed haptic system deals with vibration and heat reaction, a suitable application was designed. Interactions using the hand tracking system were carried out in the VR application. Survey and statistical experiments were used to assess whether the process of delivering tactile responses to the proposed haptic system enhances the sense of immersion and presence. In order to verify the applicability of the proposed portable hand haptic system, we confirmed whether the haptic system results in VR sickness during VR applications through the simulator sickness questionnaire [47,48]. As an experiment result, the degree of sickness was recorded to be almost negligible. It was confirmed that the proposed hand haptic system does not cause VR sickness in VR application without camera movement.

Future plans for this research are to expand the scope of tactile reactions and finger movements of the proposed portable hand haptic system so that it can be applied to numerous VR applications. Moreover, more objective analysis and verification of the system’s performance will be carried out through a comparison between the proposed haptic system and an existing haptic glove. Lastly, further research will be conducted by applying the proposed haptic system to commercial or popular VR applications in order to improve the validity of the test results. As further development of the portable haptic system that can be used in immersive VR through this process, we study portable VR feedback devices, which can be easily used by a controller or gamepad. In addition, we consistently propose a popular haptic system through portable haptic system study, which can be applied not only to a PC but also to a highly accessible mobile platform VR.

## Figures and Tables

**Table 1:** Analysis of results of test for immersion of the proposed portable haptic hand system.

Question				
**Phase**	**Standard**	**Minimum**	**Maximum**	**Mean (Standard Deviation, SD)**
(a) Appropriateness of VR application and haptic system				
Scene1	3 (Appropriate)	2.7	5	4.1 (0.61)
Scene2	3 (Appropriate)	2.5	5	3.8 (0.59)
(b) Accuracy of coincidence of object touch and vibration				
Accuracy	3 (Accurate)	2	3	2.94 (0.22)
(c) Immersion provided by vibration				
Only H	5 (Real)	1	4	2.73 (0.94)
With S	5 (Real)	3.5	5	4.43 (0.52)
Vibration Wilcoxon Result (*p*-value) H : S			$5.7605\times 10^{-5}$	
(d) Accuracy of heat reaction delivered from fingertip				
Accuracy	3 (Accurate)	1	3	2.23 (0.44)
(e) Immersion provided by heat reaction				
Only H	5 (Real)	2	4	3.09 (0.69)
With S	5 (Real)	2	5	3.32 (0.76)
Heat Wilcoxon Result (*p*-value) H : S			$9.2421\times 10^{-1}$	
Total Wilcoxon Result (*p*-value) H : S			$3.5608\times 10^{-4}$	

**Table 2:** Analysis of results of test for presence of the proposed portable haptic hand system (raw data = mean/the number of items).

		Mean (Raw Data)	Standard Deviation (SD)
Total	H	141.0 (5.88)	5.31
	S(heat)	148.2 (6.18)	6.49
	S(vibration)	150.9 (6.29)	7.92
Realism	H	41.7 (5.96)	2.10
	S(heat)	42.9 (6.13)	2.62
	S(vibration)	43.2 (6.17)	3.03
Possibility of act	H	24.5 (6.13)	1.80
	S(heat)	24.9 (6.23)	1.58
	S(vibration)	25.2 (6.30)	1.94
Quality of interface	H	18.0 (6.00)	1.18
	S(heat)	18.6 (6.20)	1.28
	S(vibration)	18.6 (6.20)	1.28
Possibility of examine	H	18.5 (6.17)	1.20
	S(heat)	19.2 (6.40)	1.17
	S(vibration)	19.2 (6.40)	1.33
Self-evaluation of performance	H	12.2 (6.10)	1.17
	S(heat)	12.0 (6.00)	1.26
	S(vibration)	12.0 (6.00)	1.41
Sounds	H	18.1 (6.03)	0.29
	S(heat)	18.3 (6.10)	1.21
	S(vibration)	19.5 (6.50)	1.25
Haptic	H	8.0 (4.0)	1.2
	S(heat)	12.3 (6.15)	0.81
	S(vibration)	13.2 (6.60)	0.96
